# Value of placental virtual magnetic resonance elastography and intravoxel incoherent motion-based diffusion and perfusion in predicting adverse outcomes of small-for-gestational-age infants

**DOI:** 10.1186/s13244-023-01503-9

**Published:** 2023-09-23

**Authors:** Jing Deng, Yuwei Cao, Yao Lu, Jiacheng Song, Aining Zhang, Meng Zhao, Xin Zhou, Xihu Mu, Feifei Qu, Feiyun Wu, Ting Chen

**Affiliations:** 1 Department of Radiology, the First Affiliated Hospital of Nanjing Medical University, Nanjing, 210029 China; 2 Department of Obstetrics & Gynecology, the First Affiliated Hospital of Nanjing Medical University, Nanjing, 210029 China; 3 MR Collaboration, Siemens Healthineers Ltd, Shanghai, China

**Keywords:** Intravoxel incoherent motion, Placenta, Small-for-gestational-age infants, Virtual magnetic resonance elastography

## Abstract

**Objective:**

It is critical to early monitor and manage small-for-gestational age (SGA) infants with truly adverse outcomes not detected by conventional methods. We aimed to explore the value of diffusion-weighted imaging (DWI)-based virtual magnetic resonance elastography (vMRE) and intravoxel incoherent motion (IVIM)-based biexponential and stretched exponential parameters in predicting adverse outcomes of SGA infants.

**Methods:**

Twenty SGA infants with adverse outcomes and forty without adverse outcomes were included in this prospective study. One DWI-based vMRE parameter [the stiffness value (*μ*_diff_)], five IVIM–based parameters [true diffusion coefficient (*D*), pseudo-diffusion coefficient (D^*^), perfusion fraction (*f*), diffusion distribution coefficient (*DDC*), and diffusion heterogeneity index (*Alpha*)] and apparent diffusion coefficient (*ADC*) were calculated and compared between groups. The predictive efficiency was compared by the logistic regression analysis and receiver operating characteristic curve analysis. The relationship between the *μ*_diff_ value with gestational age was also evaluated.

**Results:**

The placental *μ*_diff_ value was remarkably higher, and the *f*, *DDC*, and *ADC* values were considerably lower in the SGA infants with adverse outcomes compared with those without adverse outcomes. The *μ*_diff_ and *f* value were predictive risk factors for SGA infants with adverse outcomes. A combined predictive model (*μ*_diff_ and* f*) improved the predictive efficacy. Moreover, there was no statistically significant correlation between the placental stiffness value and gestational age.

**Conclusions:**

Functional MRI parameters to quantify placenta elastography and microcirculation in SGA patients. This might be a useful tool to assess placental function and a vital non-invasive supplement for predicting adverse outcomes of SGA infants.

**Critical relevance statement:**

This prospective study shows DWI-based virtual magnetic resonance elastography and intravoxel incoherent motion-based functional parameters to quantify placenta elastography and microcirculation in small-for-gestational-age patients, which could complement existing non-invasive methods for monitoring and predicting neonatal perinatal adverse outcome.

**Key points:**

• vMRE is an emerging non-invasive imaging technique for evaluating placenta stiffness.

• SGA infants with adverse outcome have stiffer placental elasticity and lower microcirculation.

• Risk factors combination displayed better efficacy in predicting adverse outcomes of SGA.

**Graphical Abstract:**

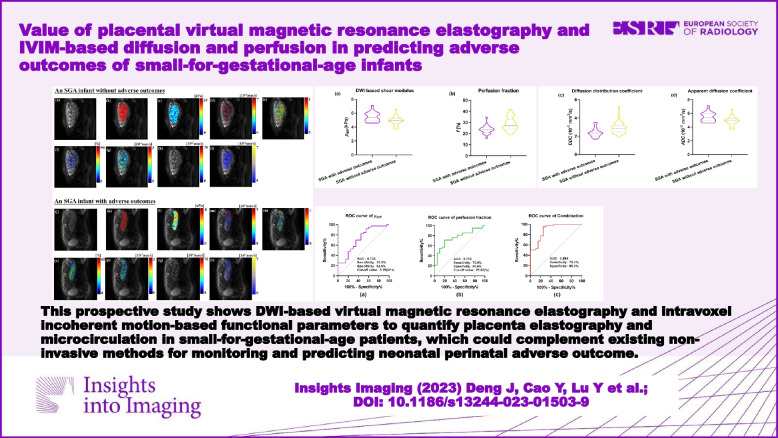

## Introduction

Small-for-gestational-age (SGA) infants are those whose neonatal birth weight is less than the 10th percentile for gestational age. SGA infants are frequently regarded as small-for-constitution but otherwise healthy infants. However, evidence indicates that certain SGA infants may have neonatal adverse outcomes such as iatrogenic prematurity, perinatal asphyxia, and low birth weight (birth weight < 3rd percentile), leading to higher infant mortality and morbidity rates [1].

The placenta is a crucial organ for fetal substance exchange, metabolism, defense, and synthesis, and its optimal functioning is a key component in determining the healthy growth of a fetus. Pathologically severe lesions in placental tissue, such as infarction and fibrosis resulting from increased placental aging and inadequate placental perfusion, can lead to poor fetal prognosis or intrauterine death [2]. We hypothesized that a subgroup of SGA infants, in fact, suffered from “stunted” fetal growth, which adapted to an inadequate nutritional environment and was undetected by conventional biophysical diagnostic methods. For SGA at-risk infants, the core of management is continuous surveillance and timely delivery [3]. Therefore, identifying this subgroup of SGA at-risk infants is crucial to predicting the adverse outcomes for pregnancies complicated by placental dysfunction.

Intravoxel incoherent motion (IVIM) is a promising approach for the quantification of biomechanical and hemodynamics information on placental tissue to assess placental function [4]. Placenta microvessel perfusion can be measured in vivo using 3 IVIM-based diffusion and perfusion parameters: perfusion fraction (*f*), true diffusion coefficient (*D*), and pseudo-diffusion coefficient (D^*^) [5]. Several studies have demonstrated that placental IVIM-based diffusion and perfusion parameters have a large potential for clinical application, but few studies have used IVIM-based functional parameters to comprehensively identify neonatal adverse outcomes of SGA infants [6, 7]. IVIM-based stretch index models could reflect tissue heterogeneity using diffusion distribution coefficient (*DDC*) and diffusion heterogeneity index (*Alpha*), which had been described in several liver-related studies [8]. Certain SGA infants with placenta dysfunction may have severe pathologic changes in the placental tissue, resulting in changes in its elasticity and stiffness. Durhan et al. used strain elastography and histologic analysis on ex vivo placentas. They revealed that the placentas in the intrauterine growth restriction (IUGR) group were stiffer than those in the normal control group, illustrating severe histopathologic alterations in IUGR [9]. The elasticity of the in vivo placentas can be detected only by shear wave elastography (SWE) because MRE is not recommended for pregnant patients. The accuracy of SWE measurement depends on the technique and experience of the operator. However, there exists a limitation in obtaining adequate measurements from the posterior placenta because of the SWE measurement depth [10, 11]. Le Bihan proposed a diffusion-weighted imaging (DWI)-based virtual elastography (vMRE) to evaluate liver fibrosis. This study demonstrated a remarkable correlation between the DWI-based shear modulus (*μ*_diff_) and MR elastographic shear modulus (*μ*_MRE_) [12]. A few studies have suggested that vMRE can be used to measure the stiffness of soft tissues [4, 13–19]. Recently, this approach was also applied to studies on the placenta [20, 21]. However, the value of vMRE for detecting the placental stiffness of SGA infants is still unknown.

In a previous study, we explored the value of IVIM in identifying very low birth infants among SGA infants [22]. The limitation was that SGA infants might also have other adverse outcomes that were not explored by us, such as neonatal asphyxia and growth restriction. Therefore, this study was aimed to unveil the predictive value of placental IVIM-based functional parameters and DWI-based elasticity in neonatal adverse outcomes of SGA infants. Besides, the relationship of the placental stiffness value from vMRE with diffusion parameters and gestational age were also evaluated.

## Materials and methods

### Study population

This prospective cohort observational study was carried out in the First Affiliated Hospital of Nanjing Medical University between January 2019 and August 2022. It was approved by the institutional ethics committee (Ethics approval number: 2019-SR-426). We successfully obtained the informed written consent from each participant.

The inclusion criteria were as follows: (1) singleton pregnancies of 28 weeks gestation or later, (2) SGA diagnosed as an estimated fetal weight < 10th percentile based on 2 consecutive ultrasonographic biometric measurements (time interval at least 2 weeks), and (3) pregnant women who underwent standard antenatal placenta magnetic resonance imaging (MRI) examination within 2 weeks of the ultrasound diagnosis of SGA.

The exclusion criteria were as follows: (1) poor-quality MRI images with severe artifacts or fetal movement; (2) maternal diseases associated with potential influence on perfusion, including pre-existing renal disease, hypertension, and diabetes mellitus; (3) placental abnormalities including placental implantation, placental previa, and placental abruption; (4) fetal genetic diseases and developmental abnormalities including structural or functional heart disease, anencephaly, and spina bifida; (5) intrauterine viral, bacterial, or parasitic infections, environmental contamination, indiscriminate use of drugs, and other harmful habits; and (6) final birth weight > 10th percentile.

**Fig. 1:**
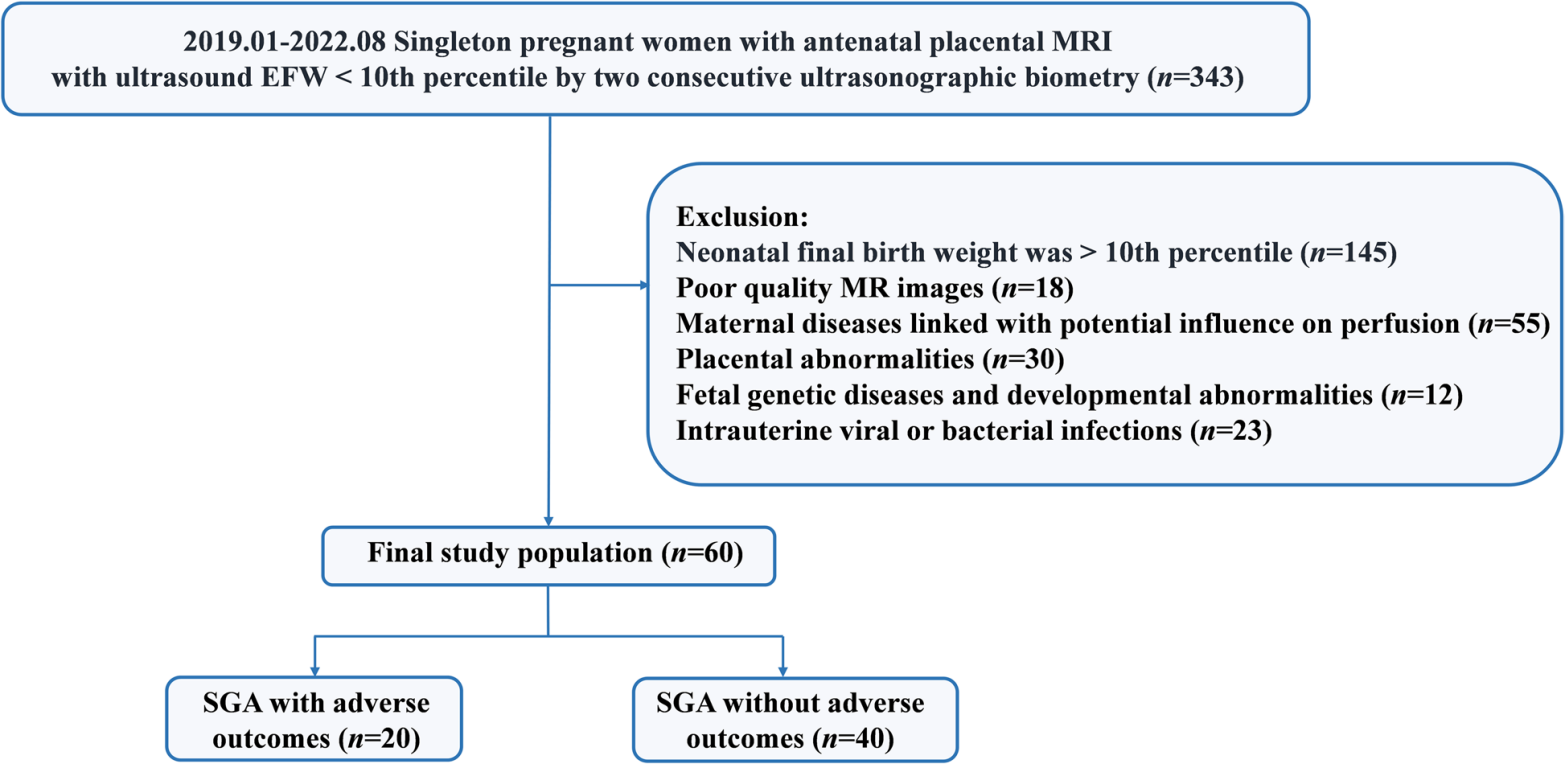
Flow diagram of the recruitment of pregnant women. EFW, estimated fetal weight; MRI, magnetic resonance imaging; SGA, small-for-gestational age

As depicted in Fig. 1, 60 patients were finally included in the study based on the aforementioned criteria.

### MRI protocol

A 1.5-T MRI system (MAGNETOM Aera, Siemens Healthcare, Erlangen, Germany) with a 12-channel surface body coil and 2 embedded channel spine coils was used to examine all patients. All patients were imaged in a supine or lateral position to ensure comfort. The MRI protocols were as follows: T1-weighted gradient echo sequence [repetition time (TR)/echo time (TE) = 6.83 ms/2.39 ms, slice thickness = 4.0 mm, field of view (FOV) = 380 × 368 mm^2^, flip angle (FA) = 70°, and in-plane resolution = 1.5 × 1.5 mm^2^]; T2-weighted half-Fourier-acquired single-shot turbo spin echo (HASTE) sequence (TR/TE = 1300 ms/167 ms, slice thickness = 4.0 mm, FOV = 380 × 309 mm^2^, FA = 70°, and in-plane resolution = 1.5 × 1.5 mm^2^); and multi-*b*-value DWI sequence (TR/TE = 6400 ms/65 ms, slice thickness = 5.5 mm, FOV = 320 × 320 mm^2^, and in-plane resolution = 3.5 × 3.5 mm^2^) with a spectrum of varying *b*-values of 0, 50, 100, 150, 200, 500, and 800 s/mm^2^. The total scan duration was less than 15 min.

### MRI data post-processing

The region of interest (ROI) of each patient was drawn with appropriate size on all continuous slices by 2 readers separately (reader 1, C.T., with 10 years of experience in fetal MRI; reader 2, S.J.C., with 5 years of experience in fetal MRI). The ROIs should contain as much of the placenta as possible while excluding regions with artificial signal loss. Both readers were blinded to patients’ information. All the ROIs of placenta DWI-based stiffness values and IVIM-based diffusion and perfusion parameters were delineated by the same method. The inter-reader repeatability was calculated using the measurements of the 2 readers. Reader 1 re-assessed all images in randomized order 1 month after the initial evaluation to evaluate intra-reader repeatability.

### Placental stiffness measurement

ROI segmentation was performed using the open-source software ITK-SNAP [23]. The stiffness value of DWI-based vMRE was determined using custom-written software in MATLAB (Mathworks, MA, USA). DWI of the lower *b*-value (*S*_low_, *b*-value = 200 s/mm^2^) and that of the higher *b*-value (*S*_high_, *b*-value = 800 s/mm^2^) were used to estimate the virtual stiffness presented by the *μ*_diff_:

1 $${\mu }_{\mathrm{diff}}=\alpha \cdot \mathrm{ln}\left({S}_{\mathrm{low}}/{S}_{\mathrm{high}}\right)+\beta$$

The scaling (*α*) and the shift (*β*) factors were set to − 9.8 and 14, respectively, according to the previous calibration studies on liver [17].

The mean values of placental *μ*_diff_ were automatically extracted from the segmented placenta regions using the custom-written software.

### Placental diffusion and perfusion parameters

IVIM-based function parameters post-processing based on the multi-*b*-value diffusion-weighted images were determined using the FireVoxel software, build 368, https://firevoxel.org/ (FireVoxel: CAL2R, New York University, NY, USA). The equation for the bi-exponential model was as follows:

2 $$S\left(b\right)={S}_{0}\left[\left(1-f\right)\cdot {e}^{-b\cdot D}+f\cdot {e}^{-b\cdot \left(D+{D}^{*}\right)}\right]$$

where *S(b)* denotes the signal intensity, *S*_0_ denotes the signal intensity for *b* = 0, *f* (perfusion fraction) denotes the volume percentage of the vascular compartment, and (*1* − *f*) represents the remaining volume fraction, tissue, or cellular compartment. D^*^ reflects the associated diffusion coefficient (pseudo-diffusion coefficient), and *D* is the diffusion coefficient (true diffusion coefficient).

The standard apparent diffusion coefficient (*ADC*) value was calculated by using a mono-exponential model with the equation:

3 $$S\left(b\right)={S}_{0}* exp\left(- b* ADC\right)$$

The *DDC* and *Alpha* value were calculated using the following stretched exponential model:

4 $$S\left(b\right)={S}_{0}* exp{\left(- b* DDC\right) }^{Alpha}$$

**Fig. 2:**
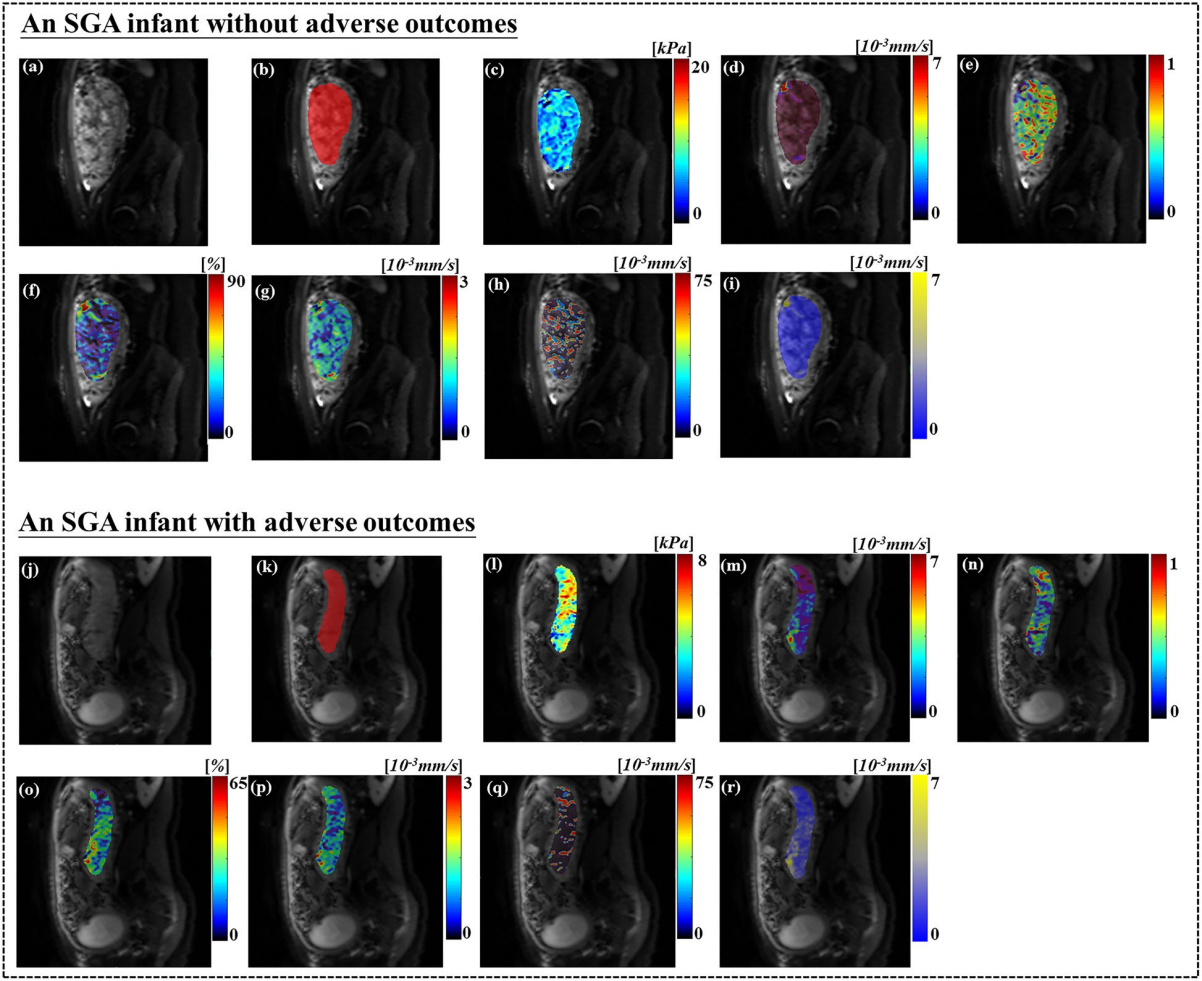
Diagram of 2 SGA infants. An SGA infant without adverse outcomes after 36.4 weeks (**a**–**i**). **a** DWI at *b* = 100 mm^2^/s depicts the placenta. **b** ROI was drawn on a diffusion-weighted image (*b* = 100 mm^2^/s). **c** DWI-based vMRE map. **d**–**h** IVIM-based diffusion and perfusion pseudo-color maps of *DDC*, *Alpha*, *f*, *D*, and D^*^. **i** *ADC* map. An SGA infant with adverse outcomes after 36.1 weeks (**j**–**r**). **j** DWI at *b* = 100 mm^2^/s depicts the placenta. **k** ROI was drawn on a diffusion-weighted image (*b* = 100 mm^2^/s). **l** DWI-based vMRE map. **m**–**q** IVIM-based diffusion and perfusion pseudo-color maps of *DDC*, *Alpha*, *f*, *D*, and D^*^. **r** *ADC* map. *ADC*, apparent diffusion coefficient; *Alpha*, diffusion heterogeneity index; *DDC*, diffusion distribution coefficient; *D*, true diffusion coefficient; D^*^, pseudo-diffusion coefficient; DWI, diffusion-weighted imaging;* f*, perfusion fraction; IVIM, intravoxel incoherent motion; ROI, region of interest; SGA, small-for-gestational age; vMRE, virtual magnetic resonance elastography

The placental DWI-based vMRE stiffness map and diffusion and perfusion pseudo-color maps are depicted in Fig. 2.

### Collection of clinical information

The clinical information, including maternal age, gestational age at MRI, neonatal birth weight, neonatal sex, route of delivery, and term or preterm delivery, was obtained from the medical records. The neonatal adverse outcomes were documented: perinatal mortality, asphyxia, neonatal birth weight < 3rd percentile, delivery term < 34 weeks, and 5-min Apgar score < 7 [24].

### Statistical analysis

The statistical analyses were performed using SPSS, version 25, for Windows (SPSS Inc., IL, USA). The inter- and intra-class correlation coefficient (ICC) was used to analyze the inter- and intra-reader agreement of the quantitative parameters. An ICC greater than or equal to 0.75 represented remarkable consistency, an ICC between 0.40 and 0.75 indicated medium consistency, and an ICC less than 0.40 represented poor consistency. Although they all exhibited remarkable agreement, the first reader’s results at the first time were used in the statistical analysis. The *t* test or Mann–Whitney *U* test was used in the statistical analysis of the continuous variables. The chi-square test was used to compare categorical variables. And multivariate logistic regression analysis was used to identify the predictive risk factors for adverse outcomes of SGA. The receiver operating characteristic (ROC) curve analysis and the area under the curve (AUC) were used to quantify and compare the diagnostic value of each remarkable parameter. The Spearman correlative analysis was performed to determine the correlation between the vMRE stiffness value and gestational age. A *p* value < 0.05 indicated a statistically considerable difference.

## Results

### Clinical characteristics

This study included 20 SGA infants with neonatal adverse outcomes and 40 SGA infants without adverse outcomes. The details of the outcomes of 20 SGA infants were as follows: perinatal mortality (*n* = 1), asphyxia (*n* = 5), neonatal birth weight < 3rd percentile (*n* = 17), delivery term < 34 weeks (*n* = 4), and 5-min Apgar score < 7 (*n* = 3). The detailed clinical characteristics of SGA infants are summarized in Table 1.

**Table 1:** Clinical characteristics of SGA infants

	SGA infants with adverse outcomes (*n* = 20)	SGA infants without adverse outcomes (*n* = 40)	*p* value
Maternal age (year)	29.90 ± 4.01	30.85 ± 3.86	.379
GA at MRI scan (week)	33.42 ± 2.76	34.25 ± 2.80	.283
GA at delivery (week)	36.31 ± 2.19	38.38 ± 1.43	.000^*^
Routes of delivery			
Vaginal delivery	4 (20.00%)	19 (47.50%)	.074
Cesarean section	16 (80.00%)	21 (52.50%)	
Preterm deliveries			.003^*^
Yes	11 (55.00%)	6 (15.00%)	
No	9 (45.00%)	34 (85.00%)	
Neonatal sex			.445
Male	6 (30.00%)	16 (40.00%)	
Female	14 (70.00%)	24 (60.00%)	
Neonatal birth weight (g)	1797.50 ± 516.43	2593.75 ± 327.81	.000^*^

*GA* Gestational age, *MRI* Magnetic resonance imaging, *SGA* Small-for-gestational age

^*^p < 0.05

### Inter- and intra-reader reliability

All parameters had remarkable consistency. The details on inter- and intra-reader ICCs are listed in Table 2.

**Table 2:** Inter-and intra-reader ICCs for the measurements of parameters

Parameters	Inter-reader ICC	Intra-reader ICC
**vMRE parameter**		
*μ*_diff_	0.811 (0.682–0.887)	0.896 (0.783–0.945)
**Diffusion and perfusion parameters**		
*f*	0.924 (0.876–0.954)	0.964 (0.940–0.978)
*D*	0.907 (0.848–0.943)	0.954 (0.916–0.974)
D^*^	0.902 (0.808–0.947)	0.937 (0.897–0.962)
*DDC*	0.958 (0.928–0.975)	0.977 (0.959–0.987)
*Alpha*	0.899 (0.837–0.938)	0.847 (0.758–0.906)
*ADC*	0.951 (0.912–0.972)	0.963 (0.932–0.979)

Data in parentheses are 95% confidence intervals

*ADC* Apparent diffusion coefficient, *Alpha* Diffusion heterogeneity index, *DDC* Diffusion distribution coefficient, *D* True diffusion coefficient, D^*^ Pseudo-diffusion coefficient, *f* Perfusion fraction, *ICC* Intra-class correlation coefficient, *vMRE* virtual magnetic resonance elastography, *μ*_diff_ Diffusion-weighted imaging–based shear modulus

### Comparison of vMRE stiffness values and IVIM parameters between SGA infants with and without adverse outcomes

**Fig. 3:**
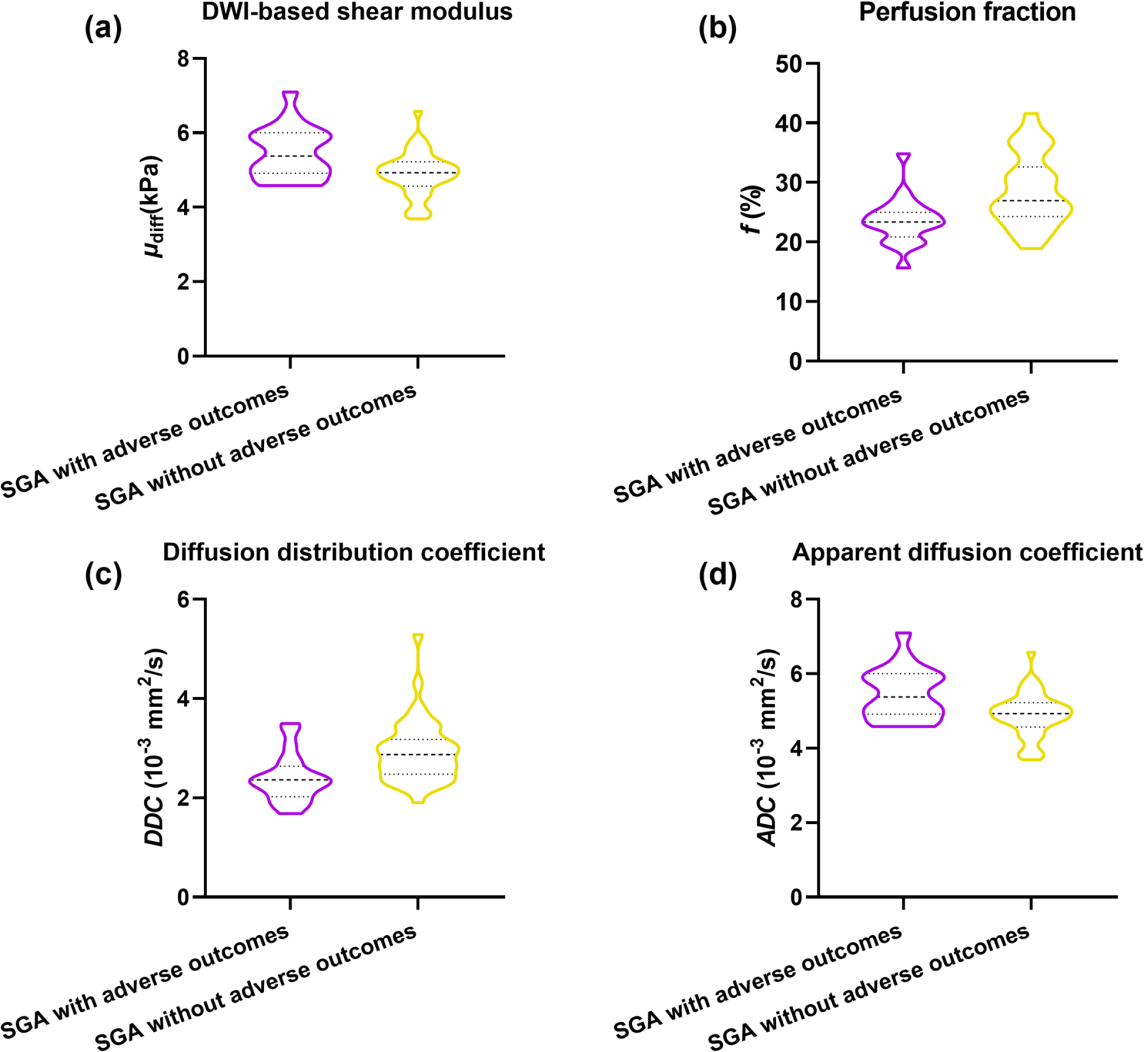
Violin plots of placental stiffness parameter (**a**) and diffusion and perfusion parameters (**b**–**d**) between SGA infants with and without adverse outcomes. *ADC*, apparent diffusion coefficient; *DDC*, diffusion distribution coefficient; DWI, diffusion-weighted imaging; *μ*_diff_, DWI-based shear modulus; *f*, perfusion fraction; SGA, small-for-gestational age

As depicted in Fig. 3 and Table 3, the *μ*_diff_ values of SGA infants with adverse outcomes were statistically substantially higher than those without adverse outcomes (*p* = 0.001). The *f*, *DDC*, and *ADC* values for SGA infants with adverse outcomes were statistically considerably lower than those without adverse outcomes (*p* < 0.05). No substantial statically difference was observed in *D*, D^*^, or *Alpha* values (*p* > 0.05).

**Table 3:** Relevant parameters comparison in the SGA infants with and without adverse outcomes

	SGA infants with adverse outcomes (*n* = 20)	SGA infants without adverse outcomes (*n* = 40)	*p* value
**vMRE parameter**			
*μ*_diff_ (kPa)	5.47 ± 0.68	4.89 ± 0.59	.001^*^
**IVIM parameters**			
*D* (10^−3^mm^2^/s)	1.55 ± 0.23	1.62 ± 0.13	.186
D* (10^−3^mm^2^/s)	161.13 ± 48.07	138.80 ± 46.58	.089
*f* (%)	23.56 ± 3.94	28.44 ± 5.98	.002^*^
*DDC* (10^−3^mm^2^/s)	2.40 ± 0.49	2.94 ± 0.63	.002^*^
*Alpha*	0.70 ± 0.04	0.69 ± 0.05	.508
*ADC* (10^−3^mm^2^/s)	2.34 ± 0.44	2.75 ± 0.45	.001^*^

*ADC* Apparent diffusion coefficient, *Alpha* Diffusion heterogeneity index, *DDC* Diffusion distribution coefficient, *D* True diffusion coefficient, D* Pseudo-diffusion coefficient, *f* Perfusion fraction, *SGA* Small-for-gestational age, *vMRE* virtual magnetic resonance elastography, *μ*_diff_ Diffusion-weighted imaging–based shear modulus

^*^p < 0.05

### Multivariate logistic regression analysis

Multivariate logistic regression analysis revealed that the *μ*_diff_ value (odds ratio [OR]: 23.446, *p* = 0.003) and the *f* value (OR: 0.590, *p* = 0.003) were predictive risk factors for adverse outcomes of SGA infants.

### Diagnostic value of vMRE and IVIM parameters in predicting the adverse outcomes of SGA infants

**Fig. 4:**
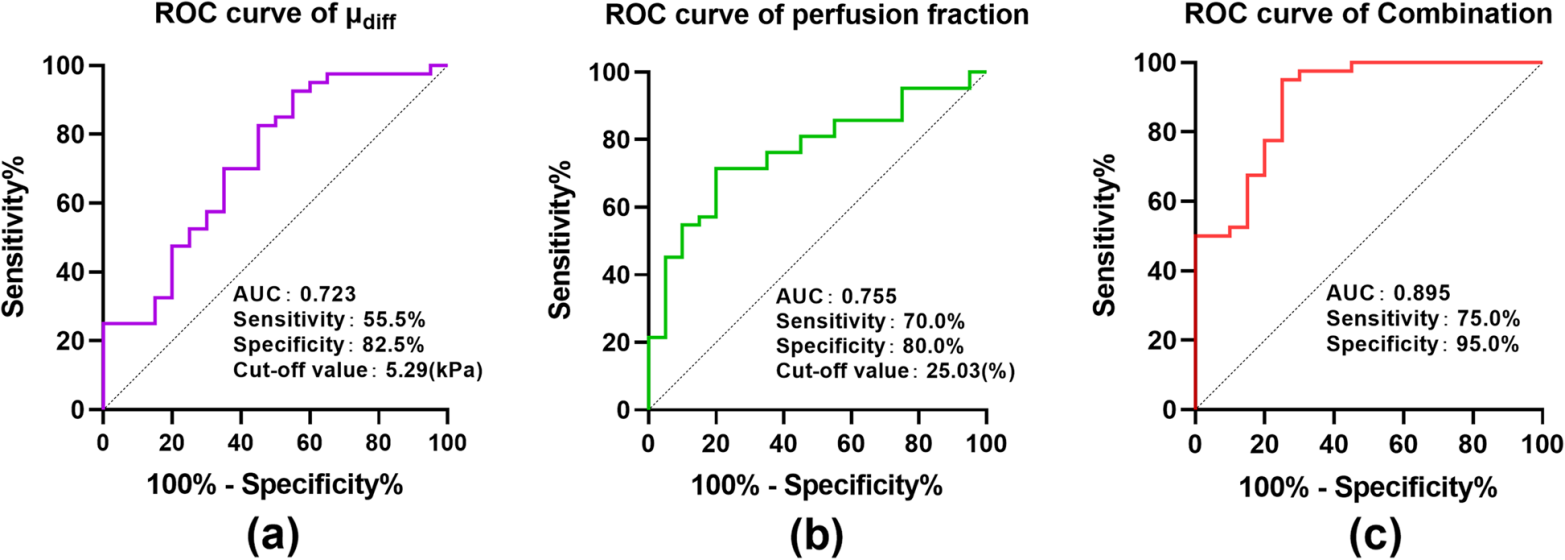
ROC curves of all the quantitative parameters. The sensitivity, specificity, cut-off values, and area under the curve for the *μ*_diff_ value (**a**), the *f* value (**b**), and the combination (**c**) are provided in each image. AUC, area under the curve; *μ*_diff_, diffusion-weighted imaging–based shear modulus; *f*, perfusion fraction; ROC, receiver operating characteristic

**Fig. 5:**
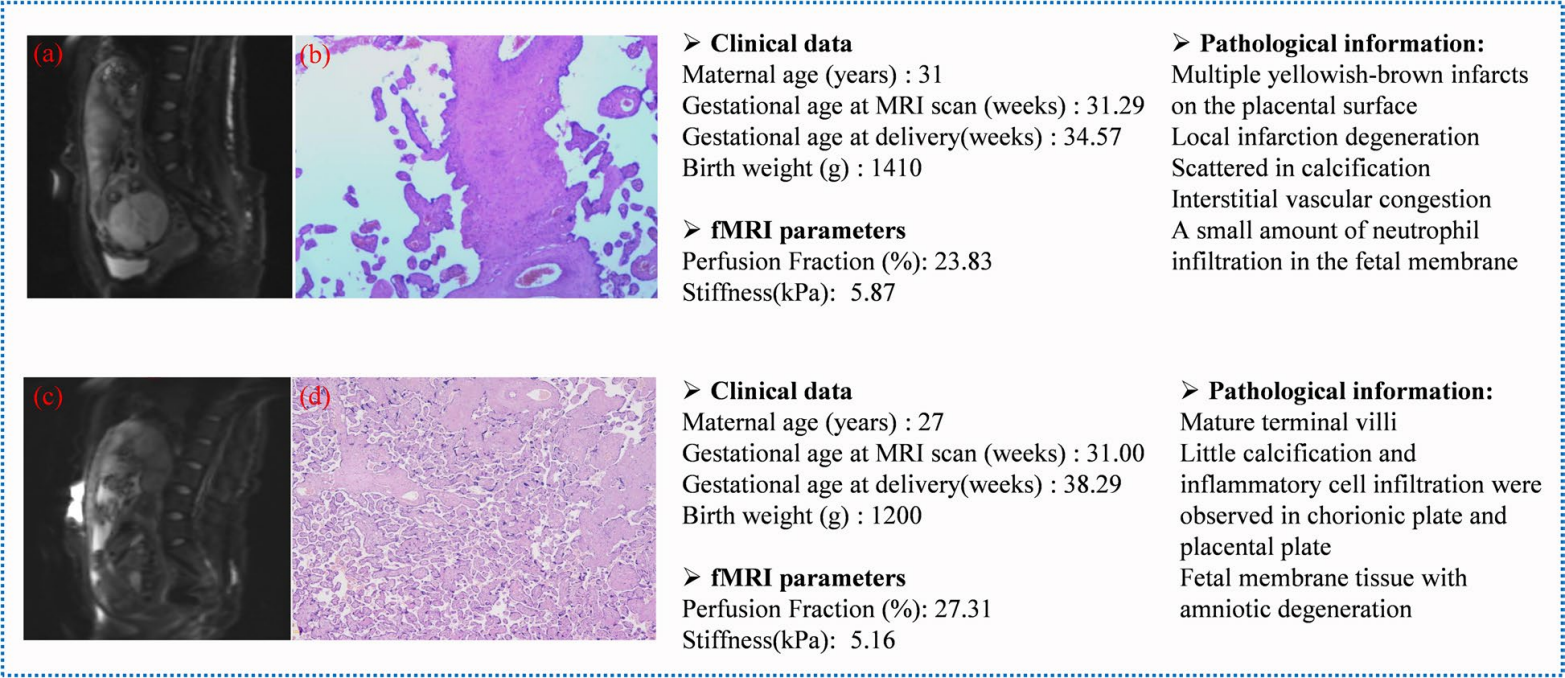
Pathologic results for 2 cases. An SGA infant with adverse outcomes (scanned after 31.29 weeks) (**a** and **b**). **a** DWI at *b* = 100 mm^2^/s. The *f* value was lower than the cutoff value (23.83% vs 25.03%) and the *μ*_diff_ value was higher than the cutoff value (5.87 kPa vs 5.29 kPa). **b** Placental pathologic information of SGA infants with adverse outcomes. An SGA infant without adverse outcomes (scanned after 31.00 weeks) (**c** and **d**). **c** DWI at *b* = 100 mm^2^/s. The *f* value was higher than the cutoff value (23.83% vs 25.03%) and the *μ*_diff_ value was lower than the cutoff value (5.16 kPa vs 5.29 kPa) (**d**) Placental pathologic information of SGA infants without adverse outcomes. DWI, Diffusion-weighted imaging; *μ*_diff_, DWI-based shear modulus; *f*, perfusion fraction; fMRI, functional magnetic resonance imaging; SGA, small-for-gestational age

Regarding the DWI-based vMRE stiffness values, the ROC curve analysis revealed that the AUC of the placental *μ*_diff_ value was 0.723 [95% confidence interval (CI): 0.582–0.863]. Regarding the IVIM-based parameters, the *f* value indicated a higher diagnostic value with the AUC of 0.755 (95% CI: 0.630–0.880). We combined the *μ*_diff_ and *f* values, which exhibited the highest AUC of 0.895 (95% CI: 0.804–0.981). The detailed results of the ROC curve analysis are displayed in Fig. 4. Two representative cases from the 2 groups are depicted in Fig. 5. As depicted in Fig. 5, the SGA infants with adverse outcomes had a lower *f* value and a higher *μ*_diff_ value. The pathologic results revealed multiple yellowish-brown infarcts on the placental surface, local infarction degeneration, and interstitial vascular congestion in the placenta. The SGA infants without adverse outcomes had a higher *f* value and a lower *μ*_diff_ value. The pathologic results revealed mature terminal villi in the placenta, little calcification and inflammatory cell infiltration, and no remarkably visible placental infarction.

### Correlation between vMRE stiffness value and gestational age

We analyzed the relationship between the stiffness value of SGA and gestational age at MRI via Pearson correlative coefficients (*r*). Both in SGA infants with and without adverse outcomes, no significant correlation was observed between the *μ*_diff_ value and gestational age at MRI (*p* > 0.05).

## Discussion

This study was novel in using a noninvasive diagnostic method named DWI-based virtual elastography and IVIM-based biexponential and stretched exponential parameters for predicting neonatal adverse outcomes of SGA infants, which benefits to compensate for the shortcomings and limitations of prenatal ultrasound. We observed that the *μ*_diff_ value was remarkably higher, and the *f*, *DDC*, and *ADC* values were considerably lower in SGA infants with adverse outcomes compared with those without adverse outcomes. Multivariate logistic regression analysis revealed that the *μ*_diff_ and *f* values were predictive risk factors. A combined predictive model (*μ*_diff_ and *f*) revealed substantially higher AUC than a single parameter, demonstrating the potential of placental vMRE elasticity and perfusion for the clinical diagnosis of SGA infants with neonatal adverse outcomes.

The vMRE used a DWI sequence to enhance the texture of tissues with dense diffusion barriers, such as fibrotic tissues [17]. Several studies have used vMRE to detect the correlations between stiffness and organ lesions. Kromrey and Le Bihan reported a correlation between vMRE and liver fibrosis [16]. Another study demonstrated the correlation between mean tissue elasticity on vMRE and surgical consistency grading in pituitary adenomas [17]. A few ultrasound reports revealed that increased placental stiffness was correlated with placental pathology [10, 25]. The present study demonstrated that the placental *μ*_diff_ values in SGA infants with adverse outcomes were remarkably higher than those without adverse outcomes, which was consistent with previous ex vivo findings [9]. And the *μ*_diff_ value was a risk factor in predicting adverse outcomes of SGA infants. It is speculated that the increased placental stiffness in SGA infants with adverse outcomes may be due to severe placental vascular dysfunction, abnormal trophoblastic structure, ischemia, and hypoxia in pregnant women [26]. These factors lead to a more severe decline in placental function and exchange dysfunction, which affects fetal nutrient metabolism and manifests pathologically as placental fibrin deposition and increased placental stiffness [27].

Some studies have demonstrated a correlation between *f* value and placental dysfunction [28–30]. For SGA infants affected by placental dysfunction, the spiral arteries in trophoblast migration could create an area with high resistance to blood flow, resulting in decreased nutrition of the intervillous space and increased vasoconstricting agent activity [26]. This results in a relative decline in the placental perfusion fraction, fetal growth restriction, and even risk of asphyxia [31]. However, previous studies mainly concentrated on one single adverse outcome of intrauterine growth restriction (IUGR) infants and also ignore SGA infants at-risk [28–30]. In this study, the *f* value, as a predictive risk factor, was lower in SGA infants with adverse outcomes than in those without adverse outcomes, suggesting that altered placental tissue hypoperfusion was one of the main pathologic changes in SGA at risk. Compared with these IVIM studies, we further revealed that this subgroup of SGA infants with a very low birth weight, a low Apgar score, and asphyxia may also have low peripheral oxygenation and perfusion. Future studies should focus on whether IVIM-based perfusion parameters can also be applied to predict fetal intrauterine distress and even the best delivery time.

The stretched exponential model is another new method to fit the apparent diffusion attenuation characteristics that reflect a continuous distribution. The *DDC* value reflects the diffusion motion of water molecules like the *ADC* value and was considered a surrogate marker of cellular density [8, 31]. The dysfunctional placenta of SGA infants potentially leads to sparse villi compared with normal placental tissue, increased interstitial hyperplasia and necrosis, marked reduction of intravascular vessels within the villi, and narrowing or occlusion of the lumen [26, 32]. These changes in placental tissue microstructures lead to restricted diffusion of water molecules, which was proved by the reduced *DDC and ADC* values in SGA infants with adverse outcomes reported in our study. However, compared with the *μ*_diff_ and *f* values, the *DDC and ADC* values were not the independent predictors of risk factors. This might be related to the fact that the *DDC and ADC* values fail to exclude the effect of microcirculation perfusion [33]. *Alpha* reflects the complexity of the tissue microstructure [8]. In our study, we found that the *Alpha* value is similar between SGA with and without adverse outcomes, which is consistent with previous reports about hepatic fibrosis [8]. We cautiously speculated that increased intraplacental infarction and decreased microcirculation perfusion might not contribute to the increased heterogeneity of placental tissue [8, 33]. Despite no statistically difference in *D* values between the groups of SGA infants with and without adverse outcomes, the *D* values of SGA infants with adverse outcomes were slightly lower than those of SGA infants without adverse outcomes [1.55 ± 0.23 (10^−3^ mm^2^/s) vs 1.62 ± 0.13 (10^−3^ mm^2^/s), *p* = 0.186], which could be due to the small sample size. Future studies with larger sample size may clarify the potential clinical value of the *D* value in SGA infants at risk for early diagnosis, early management, and improvement of prognosis.

In addition, we found that there was no significant correlation between the *μ*_diff_ value and gestational age at MRI, which was in ordinance with reported ultrasound studies [34, 35]. However, Liu et al. found the stiffness value of placenta was the lowest at 26 weeks and showed an upward trend from 26 to 36 weeks [21]. The contradiction between our results could be attributed to various factors, including the individual differences, as well as the differences of gestational age included in the study population selection between us and Liu et al. The relationship between *μ*_diff_ and gestational age needs to be further investigated with a larger sample size of SGA infants.

## Limitations

This study had several limitations. First, the study population was small. Further multi-center, large-sample studies are needed to validate the findings. Second, although we demonstrated that IVIM-based functional parameters might correspond to placental pathologic findings, the pathologic examination was not performed for the placenta of all patients, especially those without adverse outcomes. Third, the *α*, *β*, and key *b*-values (200 and 800 s/mm^2^) for calculating the stiffness values on DWI-based vMRE were derived from the studies without calibration on placenta [20, 21]. Therefore, the results obtained in this study were preliminary. The calibration studies using SWE are the next step to explore the optimal parameters for calculating placental elasticity.

## Conclusions

In conclusion, placental IVIM-based diffusion and perfusion and DWI-based vMRE potentially provide a novel tool for detecting the adverse outcomes of SGA infants. Therefore, further studies should be conducted to determine the best calibration parameters and MR protocols, specifically for the placenta, so as to provide more accurate assistance to clinical treatment.

## Data Availability

The datasets generated and/or analyzed during the current study are not publicly available due to PACS system regulated by the First Affiliated Hospital of Nanjing Medical University but are available from the corresponding author on reasonable request.

## Abbreviations

EFW: Estimated fetal weight
IUGR: Intrauterine growth restriction
IVIM: Intravoxel incoherent motion
MRI: Magnetic resonance imaging
ROI: Region of interest
SGA: Small-for-gestational-age
SPSS: Statistical Product and Service Solutions
SWE: Shear wave elastography
vMRE: Virtual magnetic resonance elastography
